# Missense3D-TM: Predicting the Effect of Missense Variants in Helical Transmembrane Protein Regions Using 3D Protein Structures

**DOI:** 10.1016/j.jmb.2023.168374

**Published:** 2023-12-07

**Authors:** Gordon Hanna, Tarun Khanna, Suhail A. Islam, Alessia David, Michael J. E. Sternberg

**Affiliations:** Centre for Integrative Systems Biology and Bioinformatics, Department of Life Sciences, Imperial College London, London SW7 2AZ, UK

**Keywords:** transmembrane, variants, prediction, protein structure, disease

## Abstract

Variant effect predictors assess if a substitution is pathogenic or benign. Most predictors, including those that are structure-based, are designed for globular proteins in aqueous environments and do not consider that the variant residue is located within the membrane. We report Missense3D-TM that provides a structure-based assessment of the impact of a missense variant located within a membrane. On a data-set of 2,078 pathogenic and 1,060 benign variants, spanning 711 proteins from 706 structures, Missense3D-TM achieved an accuracy of 66%, Mathews correlation coefficient of 0.37, sensitivity of 58% and specificity of 81%. Missense3D-TM performed similarly to mCSM-membrane: accuracy 66% vs 61% (*p* = 0.02) on an unbalanced test set and 70% vs 67% (*p* = 0.20) on a balanced test set. The Missense3D-TM website provides an analysis of the structural effects of the variant along with its predicted position within the membrane. The web server is available at http://missense3d.bc.ic.ac.uk/.

## Introduction

Transmembrane proteins are central in many cellular processes, such as transportation, signalling and adhesion,^1^ and include important classes of drug targets such as the G-protein Coupled Receptors (GPCRs).^2,3^ Missense variants in transmembrane proteins are associated with many conditions, such as cardiovascular diseases and carcinogenesis.^4^ Consequently, identifying changes to structural features present in proteins can aid in predicting whether missense variants are pathogenic or benign. Although sequence-based predictors provide accurate results, they often produce a binary output or numeric score related to the probability of pathogenicity without an accompanying explanation of the underlying structural effects.^5,6^ A structural analysis can provide information on how a missense variant disrupts a protein structure and/or function, thus assisting in understanding which biological mechanisms may be affected and informing further study. However, the majority of current approaches for predicting the effects of missense variants are not tailored for residues in transmembrane regions because they do not take into account the different physico-chemical conditions of the membrane lipid bilayer compared to that in globular proteins.^4,7,8^

There are only a few methods designed for transmembrane proteins that model the variant structure and assess the impact of the change in the context of the residue being within the membrane. mCSM-membrane^9^ integrates structural and sequence-based information using a graph-based approach to represent extracted features such as molecular interactions and pharma-cophores and uses the random forest with extra trees method to provide supervised learning. Boroda-TM^10^ uses data from multiple sources including sequence data, structure data and energy data, but only provides predictions if an experimental structure is available. MutTMPredictor^11^ is an ensemble method which combines sequence and structure-based features including energy data and the results of other predictors.

Here we present Missense3D-TM which has been developed to assess the impact of missense variants based on their effect upon a protein structure. Missense3D-TM is an extension of our variant predictor Missense3D which was developed for globular proteins.^12^ A web server implementing Missense3D-TM is available at http://missense3d.bc.ic.ac.uk/.

## Algorithm Development

### Datasets

**Figure 1:**
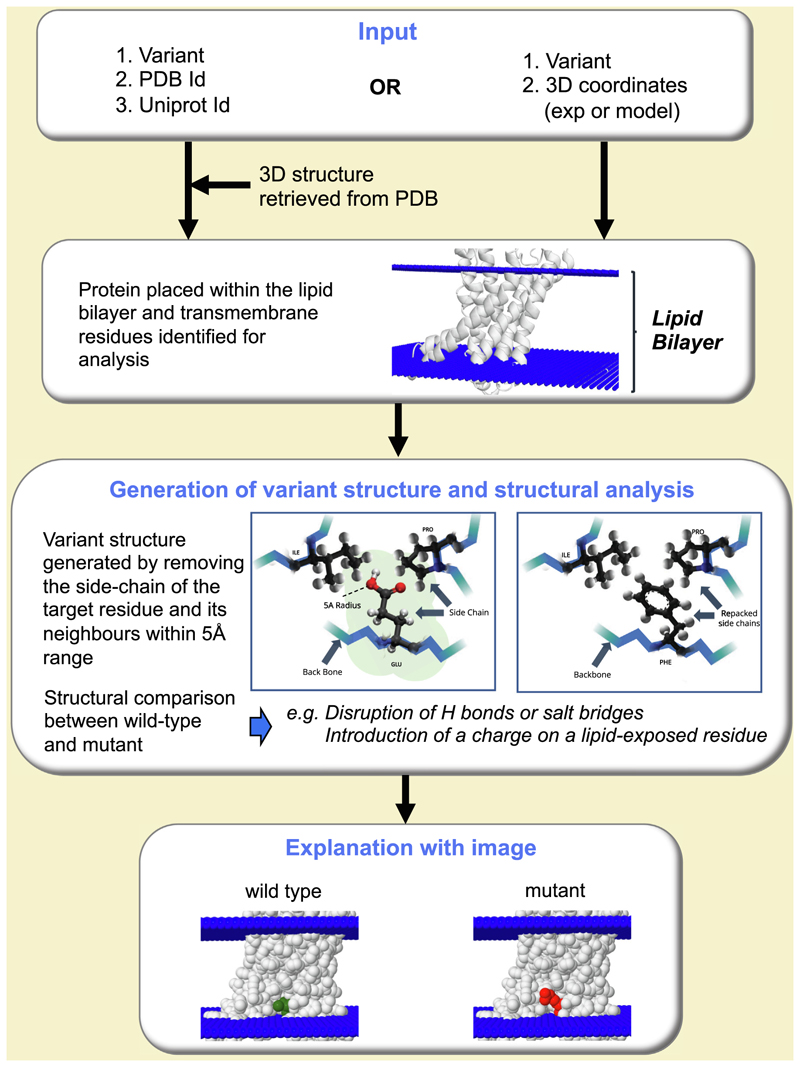
The Missense3D-TM algorithm. The pipeline utilized by the Missense3D-TM algorithm for the analysis and interpretation of the structural effect of amino acid substitutions in transmembrane regions is presented.

An overview of the Missense3D-TM algorithm is presented in Figure 1. To develop and benchmark Missense3D-TM, a dataset of 5,242 transmembrane human proteins was extracted from UniProt (June 2019 release). We ensured that between the training and testing datasets no protein regions shared more than 40% identity (see Supplementary material). The 40% sequence identity cut off was a compromise between removing all homologues between training and testing and providing sufficient variants for the development and benchmarking of the algorithm. Coordinates corresponding to transmembrane regions were available for 1,015 proteins. 316,294 variants for the 1,015 transmembrane proteins were retrieved from our in-house variant database Missense3D-DB^13^ (see Supplementary Table S1). Variants with conflicting or no phenotypic interpretation were excluded. The remaining 10,597 variants were annotated as “pathogenic” or “benign”. In order to increase the benign dataset, variants with no pathogenic or uncertain annotation and with minor allele frequency (MAF) > 0.01 were included in the benign dataset. These 10,597 variants were then filtered to retain only those identified by UniProt and Missense3D-TM (as described below) as located within a transmembrane region (i.e. within the lipid bilayer). The final dataset included 3,138 variants (2,078 pathogenic and 1,060 benign) harboured by 711 proteins. A total of 706 structures (78 experimental and 628 modelled) were used. 696 variants (22% of 3,138) were mapped to an experimental structure of which 535 variants were pathogenic (26% of the 2,078 pathogenic total) and 161 variants were benign (15% of the 1,060 benign total) see Supplementary Table S1.

### Identification of transmembrane residues

Transmembrane regions are identified using an in-house software (TM-placement) which is based on the pseudo-energy potential approach (Ez potential)^14^ for protein insertion in lipid membranes. The first step of TM-placement is to translate the coordinates of the protein so that its centroid is located at the origin of the coordinate system. TM-placement then orients the protein with respect to a dummy lipid bilayer with a hydrophobic thickness of 30 Å (oriented along Z-axis) by assessment over three degrees of freedom; rotation about the X-axis (0–360), rotation about the Y axis (0–360) and translation along the Z-axis which is perpendicular to the bilayer (−30 to 30). For each orientation the transfer energy is calculated and the orientation with the minimum energy is selected as the preferred orientation. The default mode which is executed with Missense3D-TM uses a rotation step size of 18° and z translation step size of 3.0 Å which calculates transfer energy at 8,400 distinct protein orientations and z-axis positions with respect to the lipid bilayer. We assessed the performance of TM-placement in identifying whether a residue was within the membrane taking the OPM database as the ground truth and showed that, on a dataset of 187 structures, TM-placement exhibited 87% sensitivity, 91% specificity and 89% accuracy for the location. The Supplementary Material (Figure S1) provides distributions of these results on a per protein basis and shows that for nearly all helical proteins TM-placement closely reproduces the OPM assignment although initial results show the possibility that beta-barrels may not be successfully placed within the bilayer.

### Generation of variant structures

The approach follows that of Missense3D^12^ for globular proteins and Missense3D-PPI^15^ for variants at protein interfaces. For each variant, a structure was generated from the wild-type coordinates using SCWRL4.^16^ Briefly, the side chain of the target residue (residue to be substituted) and the side chains of any residue within 5 Å from the target residue (defined by any pair of inter-residue atoms closer than 5 Å) were removed from the coordinates. The side chain of the target residue was replaced with the variant side chain, the wild-type side chains of the neighbouring residues reintroduced and then repacked using SCWRL4^16^ to generate the coordinates of the variant.

### Evaluation of the structural impact of the variant

The metrics used to evaluate the performance of the algorithm are given in Supplementary Material. The algorithm evaluates if the variant structure has at least one unfavourable stereochemical feature, such as breakage of a disulphide bridge.

A major addition beyond Missense3D is that some features assessed by Missense3D only for buried residues in a globular protein, such as replacing a nonpolar side chain by a charged residue, are now applied to any residues in the transmembrane region whether buried or exposed in the structure. In addition, any charged residue in either of the lipid headgroup regions (defined as 2.0 Å beyond the bilayer), which could have a role in the orientation of the protein relative to the bilayer, is examined. Any substitution from a charged to an uncharged residue or switch in charge within this region can be potentially structurally damaging. As in Missense3D, if one or more stereochemical features are found to be disrupted in the variant structure, the program predicts the variant as pathogenic.

### Evaluation of Missense3D-TM

The performance of Missense3D-TM was compared to Missense3D, which was developed to assess the effect of missense variants in globular monomeric proteins (Table S2). Missense3D-TM outperformed the standard Missense3D algorithm on the testing set in terms of overall accuracy which increased from 53% to 66% (*p* < 1 x 10^-10^, two-tailed McNemar’s test). The Mathews correlation coefficient (MCC) improved from 0.27 to 0.37. Sensitivity improved from 35% to 58%, however specificity was reduced from 90% to 81%. We analysed whether Missense3D-TM performed differently on experimental vs homology derived models (Tables S3 and S4). This analysis showed that Missense3D-TM performed better on experimental structures than models: accuracy 83% vs 68%, MCC 0.62 vs 0.37 on a balanced dataset (Table S4).

The performance of each structural category assessed by Missense3D-TM on the unbalanced training and test datasets is presented in Supplementary Figures S2 and S3). In the test dataset, two categories (Buried_Charge_Switch and Buried_Glycine_In_Bend) produced no false positives and hence had infinite TPR/FPR ratio, though the numbers of variants involved were very small (*n* = 3 and *n* = 1 respectively). Aside from these, the category with the highest TPR/FPR ratio (TPR/FPR = 7.47) was TM_Salt_Bridge_Broken (breakage of a salt bridge exposed to the transmembrane region). The strong performance for this category is in accordance with the stabilising effect of salt bridges within the transmembrane region.^8^ The category which correctly identified the highest number of damaging variants (n = 90) was TM_Charge_Introduced (introduction of a charged residue exposed to the transmembrane region). Again, this is in keeping with the recognised deleterious effect of placing an unbalanced charged residue within the hydrophobic lipid bilayer ^17^.

In the testing dataset, only a single variant was identified by the TM_Charge_Switched category and no variants were identified by the TM_HBonds_Broken, CIS_Proline_Replaced and Disulphide_Bond_Broken categories. While CIS proline residues occur only rarely, it is known that when they do occur and are mutated, they are likely to result in a damaging structural change and hence this category was retained in Missense3D-TM. Similarly, inter-helical hydrogen bonds are important for the stability of transmembrane proteins ^17^ and we have therefore retained the TM_HBonds_Broken category as it performs well when detected. The remaining two categories, TM_Charge_Switched and Disulphide_Bond_Broken, were also retained due to their good performance on the training dataset.

Our testing and training datasets contained more pathogenic variants than benign (ratio 1.9:1) thus raising the possibility of a bias towards damaging predictions. To exclude this, we sub-sampled our datasets to produce balanced datasets. Sampling was by random selection of pathogenic variants within proteins while retaining variants from as many different proteins as possible. Only 256 out of the 264 benign variants could be processed using mCSM-membrane and this balanced test dataset (256 benign and 256 damaging variants) was used for comparison across all 3 predictors (Tables S5 and S6). On this balanced testing dataset (256 benign and 256 pathogenic variants) Missense3D-TM outperformed the standard Missense3D algorithm in terms of overall accuracy which increased from 63% to 70% (*p* < 1 x 10^−10^, two-tailed McNemar’s test). The Mathews correlation coefficient improved from 0.30 to 0.40. Sensitivity improved from 35% to 59%, however specificity was reduced from 90% to 80% (Table S5).

The performance of Missense3D-TM was compared to mCSM-membrane^12^ which also uses structural features to predict the effect of variants within transmembrane proteins (Table S2). On the unbalanced test set, Missense3D-TM achieved accuracy 66% versus 61%, (McNemar *p* = 0.02); sensitivity (58% vs 52%), specificity (81% vs 81%) and MCC (0.37 vs 0.31). However, on the balanced testing dataset of 512 variants, both algorithms recorded slightly higher scores with Missense3D-TM versus mCSM-membrane results showing accuracy 70% versus 67%, (McNemar *p* = 0.20); sensitivity (59% vs 53%), specificity (80% vs 81%) and MCC (0.40 vs 0.36). The lack of a significant difference between Missense3D-TM and mCSM-membrane probably results from a combination of the smaller number of variants and the lower proportion of pathogenic variants which are particularly well predicted by Missense3D-TM. We were unable to compare Missense3D-TM to Boroda-TM as their website only provides precalculated results on their selected set of experimental coordinates.

A comparison was carried out between Missense3D-TM performance on variants in the CORE vs HEAD locations from the unbalanced test dataset. This showed slightly higher MCC for variants located in the CORE. However, the number of variants identified as being in the HEAD region was a relatively small proportion of the dataset (*n* (HEAD) = 27, *n* (CORE) = 767, see Table S7).

Finally, we also considered how the performance of the Missense3D-TM algorithm varied with the number of TM segments present in the protein harbouring the variant. It was difficult to draw reliable conclusions in some groupings due to the small numbers of variants present but the accuracy reported for all categories was 55% or above except for 2 cases (5 TM’s and 15 TM’s) where the number of variants was low (*n* (5TM) = 2 and *n*(15TM) = 11, see Table S8).

### The Website

The Missense3D-TM algorithm is freely available at http://missense3d.bc.ic.ac.uk/. There are two ways in which a user may specify a query: (1) by supplying a UniProt ID and a residue position (numbering is according to UniProt amino acid sequence); (2) by supplying a coordinate file and a residue position according to the PDB sequence numbering. In both cases the chain identifier and wild-type and mutant amino acids must also be specified. The query input page contains example queries to help guide users as to what kind of input is expected.

**Figure 2:**
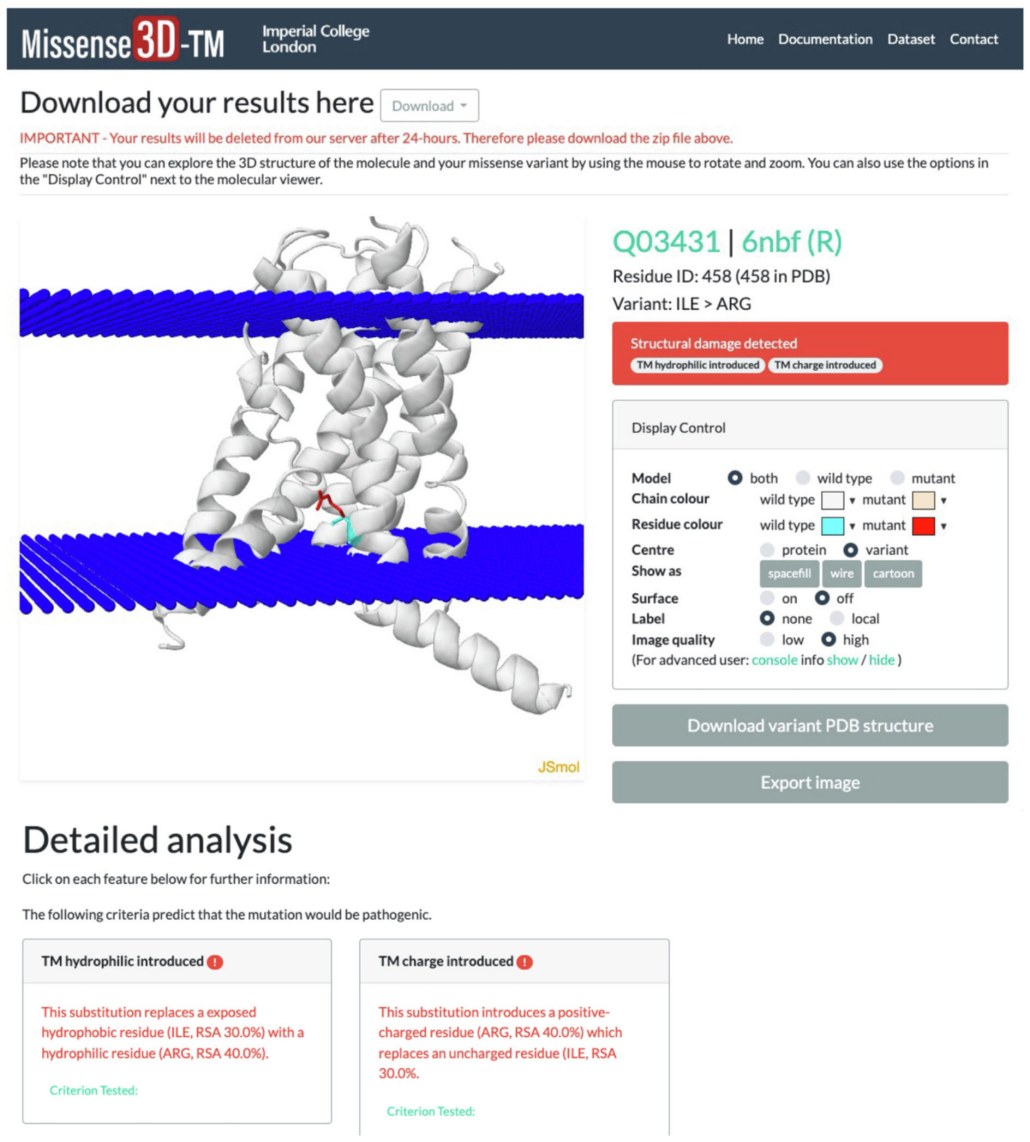
The Missense3D-TM results page. The Missense3D-TM results page details the results of the structural analysis and allows visualization of the wild type and mutant structures.

The Results summary page provides a brief report of any predicted structural change that is identified as damaging and contains a link to a detailed Results page which is shown in Figure 2. This detailed Results page includes an interactive JSmol structure display depicting the wild-type and mutant residues located on the protein structure. The image also shows the predicted location of the protein within the lipid bilayer. Display options are provided along with a JSmol command interface which supports further control. Further down the page, the next section shows a detailed account of each structural change identified as damaging. Below this is a section showing all other structural features analysed but not identified as damaging. Finally, any change in hydrogen bonds that was identified between wild-type and variant structure irrespective of whether it met our criterion for hydrogen bond breakage is reported in a pop-up display at the end of the page for completeness since these may represent borderline cases that merit further manual investigation.

The main areas of display in the detailed results web page include the JSmol structure viewer and show the location of the variant with respect to the lipid bilayer. The display also provides a summary description of the variant residue and the structural features identified as damaging together with the remaining features which were analysed and predicted to be tolerated.

As a general indication of the time required to process variants using the Missense3D-TM website, two randomly sampled variants within 7-spanning transmembrane proteins were timed and took approximately 1 minute and 15 seconds (1 minute and 10 seconds for variant p.A227V in protein Uniprot id O15529 and 1 minute 15 seconds for p.L467P in protein Uniprot Id P16473). By contrast two randomly selected single-spanning proteins were also processed via the website taking approximately 30 seconds for the first (Uniprot Id P15529 variant p.A353V) and approximately 2 minutes for the second (Uniprot Id Q15399 variant p.V587G). The longer processing time for the latter indicates that this varies with the size of the protein rather than the number of TM-spanning segments.

### Case Studies

**Figure 3:**
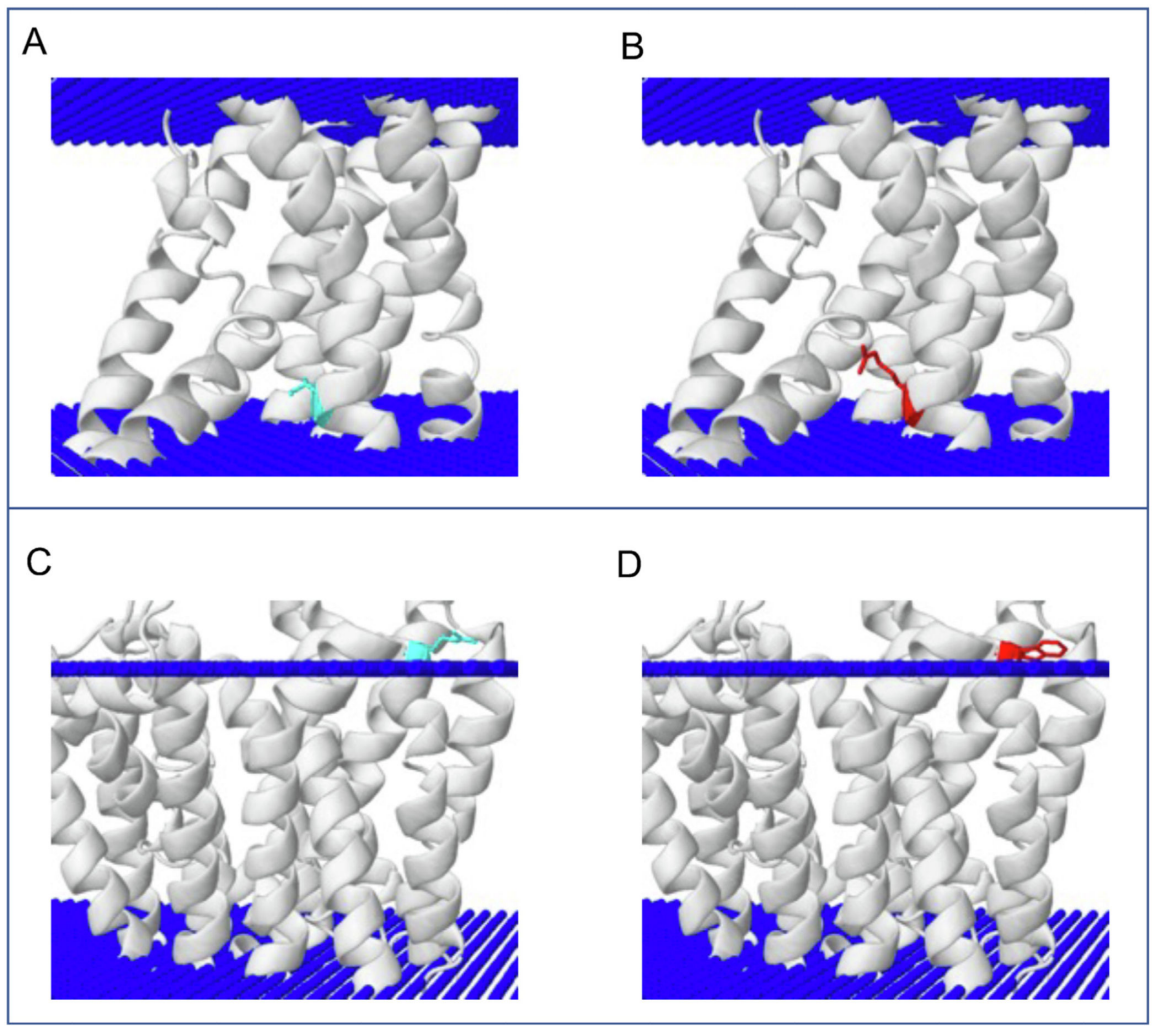
Case studies. Variant p.I458R in PTH1R (PDB Id 6nbf): the wild type residue isoleucine is presented in cyan in panel A, whereas arginine is presented in red in panel B. Variant p.R93W in SLC2A1 (PDB Id 5eqg): the wild type residue arginine is presented in cyan in panel C, whereas tryptophan is presented in red in panel D. In all figures, the lipid bilayer is presented as blue spheres.

Missense variant p.I458R (dbSNP id rs121434600) in the parathyroid hormone receptor (PTH1R, Uniprot Q03431) causes the rare skeletal disorder metaphyseal chondrodysplasia.^18^ Isoleucine 458 is located in the protein transmembrane region and is exposed to the lipid bilayer (PDB 6nbf, chain R, residue position 458 on 6nbf). Its substitution to arginine is predicted damaging by Missense3D-TM because of the introduction of a hydrophilic charged residue. The same missense variant is predicted tolerated by Missense3D for globular proteins (Fig 3 panel A and B).

The second case study is variant p.R93W (dbSNP Id rs267607061) in the glucose transporter solute carrier family 2 (SLC2A1, Uniprot id P11166), which causes the rare neurological disorder paroxysmal exertion-induced dyskinesia.^19^ This variant is predicted damaging by Missense3D-TM because of the loss of a charged residue (“charge replaced”) in the polar head region of the transmembrane protein region (PDB, chain A, residue position 93 on 5eqg). This variant is predicted tolerated by Missense3D (Fig 3 panel C and D).

## Discussion

The Missense3D-TM algorithm builds on the Missense3D concept and new transmembrane-specific criteria intended to address the different chemistry of the lipid bilayer. For cytosolic and extracellular variants located on a transmembrane protein the analysis should be performed using our standard Missense3D predictor.

Tailoring the Missense3D-TM analysis criteria to the general properties of transmembrane regions has resulted in a significant improvement in the sensitivity, accuracy and MCC score of the algorithm compared to our standard Missense3D although we observed a reduction in specificity. Predicted protein structures as well as experimentally-determined coordinates can be input into Missense3D-TM. Missense3D^12^ was designed to be applicable to both experimentally-determined and predicted structures and deliberately adds 1 Å to standard atom–atom distances for salt-bridge, disulphide bonds and hydrogen bonds and the other structural features typically are designed not to be highly sensitive to precise atomic positions. We have shown that Missense3D has a similar performance for models^20^ and experimental structures. Although Missense3D-TM has also been developed to work well with 3D models, its prediction accuracy is higher for experimental structures than for models. As our dataset is heavily biased towards models (*n*(models = 628), *n*-(experimental = 78)) we expect that performance on a dataset containing roughly equal numbers of experimental and modelled structures would be higher than reported for our testing dataset. The capacity to analyse 3D-models is particularly important given that there are so few experimental structures available due to the difficulties involved in isolating transmembrane proteins from the lipid bilayer while maintaining their structural stability. Missense3D-TM also supports the use of AlphaFold models.^21,22^ However, caution should be used when using AlphaFold models and users should examine the accuracy measures provided (predicted local distance difference test (pLDDT) and the predicted aligned error (PAE) matrix) in order only to use confidently predicted structures.^23,24^

Missense3D-TM assumes that all transmembrane regions have the same composition although it has been shown that membrane thickness and composition may vary for different types of cell membranes. Moreover, the head region near the membrane boundary exhibits different properties from the rest of the intra-membrane environment. Although Missense3D-TM does only apply a subset of criteria to variants identified as being within the head region, further specialisation could be developed for this region and also for accommodating differences between different transmembrane environments.

Currently Missense3D-TM predictions are based on the identification of a structural damage within the single protein chain. There are alternative explanations for why a variant may have a pathogenic effect without exhibiting structural changes. Functional effects, binding area effects, disruption of post-translation modification sites and allosteric effects are just a few examples ^25^. This may account for some of the false negatives detected by Missense3D-TM.

Missense3D-TM was developed using human transmembrane proteins which are predominantly alpha helices. Only one beta barrel was present in our protein dataset and its placement within the lipid bilayer was not accurate. None of the variants analysed were harboured by beta barrel proteins and, at present, we do not recommend using Missense3D-TM for beta barrel proteins.

In conclusion, we have developed Missense3D-TM, an algorithm tailored for the prediction of variants within transmembrane proteins that provides a structure-based explanation for the predicted impact of the variant.

## Supplementary Material

SI
