# Supplementary material for: Missense3D-TM: Predicting the Effect of Missense Variants in Helical Transmembrane Protein Regions Using 3D Protein Structures
Source: J Mol Biol. Author manuscript; Available in PMC 2025 Mar 24. (PMC7617522; doi:10.1016/j.jmb.2023.168374)
Supplement: SI [file EMS203543-supplement-SI.pdf]

Supplementary Information

Phyre2.2: A community resource for template-based protein structure prediction

Harold R Powell, Suhail A Islam, Alessia David & Michael J E Sternberg +

Centre for Integrative Systems Biology and Bioinformatics
Department of Life Sciences
Imperial College London
London SW72AZ, UK

+ m.sternberg@imperial.ac.uk

Sorting the list of hits

The approach is that matches are first identified where the HHBlits E-value <= 0.001. These are ranked in order of increasing values of R where:

R = P x Nalign x ID

where P is the probability from HHBlits, Nalign is the number of aligned residues and ID is the % identity (with target sequence over aligned region). Then the rank ordered list is progressed down and if the next domain in the list has a pairwise coverage of more than 90% of its residues overlapping with any other they are placed in a single cluster which is extended to include all residue positions in contributing domains. This procedure is repeated as one progresses down the list comparing each domain hit with the extended region in each cluster. Within each cluster the best example is selected based on a rank R’ where

R’ = Nalign x ID

The highest rank domain in each cluster is moved to the top of the new list; the order of all other solutions is retained from the original list.

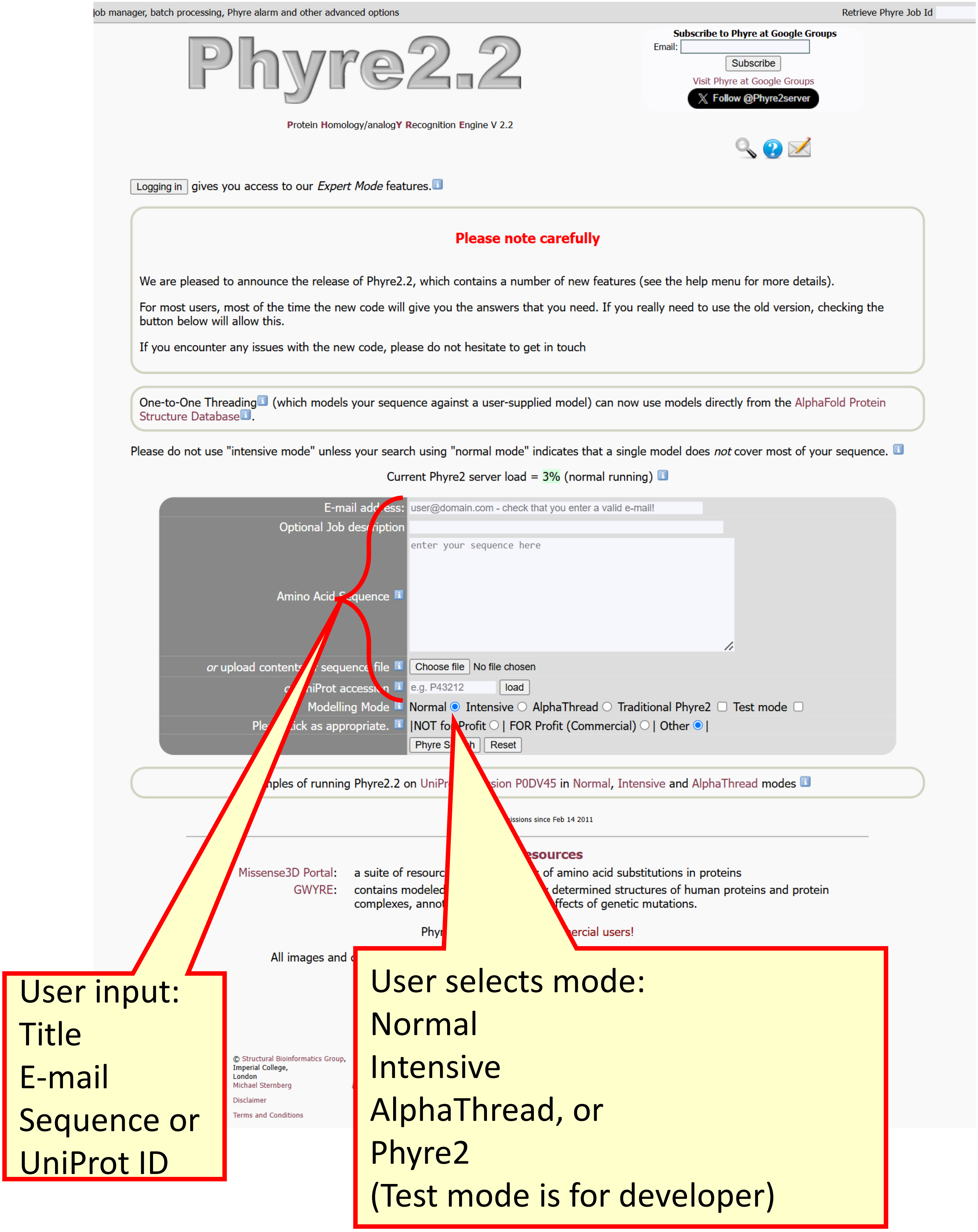

Figure S1 – The landing and input page for Phyre2.2

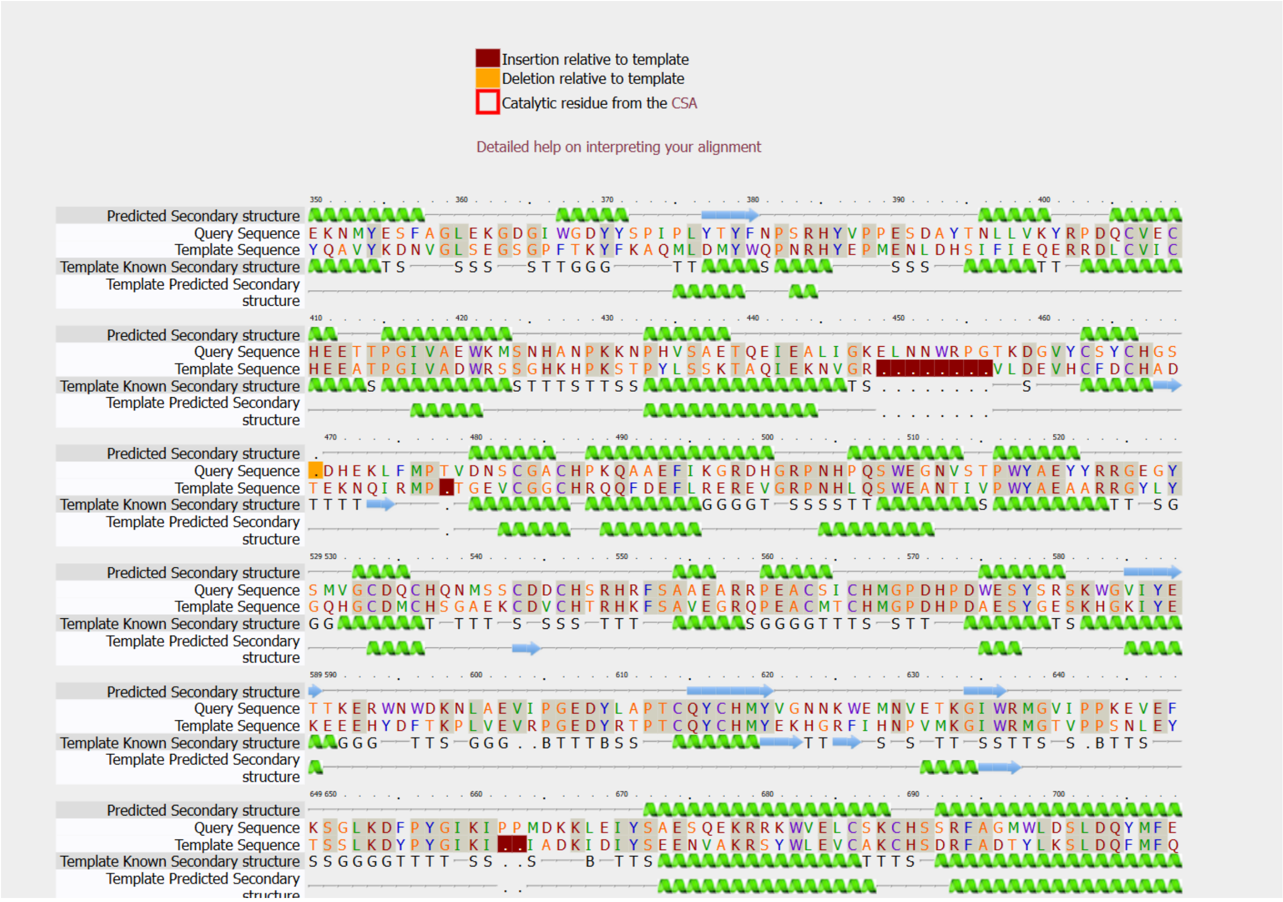

Figure S2 – Screenshot of the alignment. The sequences of the query and template are shown. Green spirals represent α-helices and blue arrows β-sheet strands.

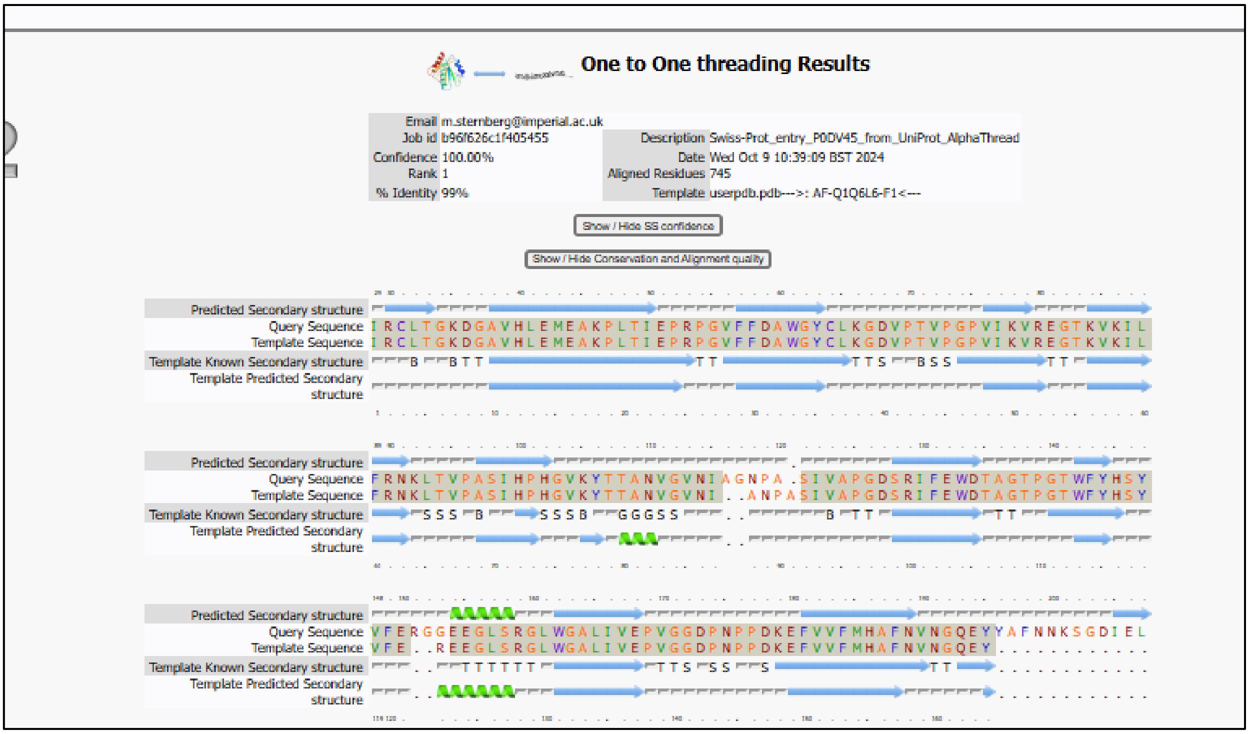

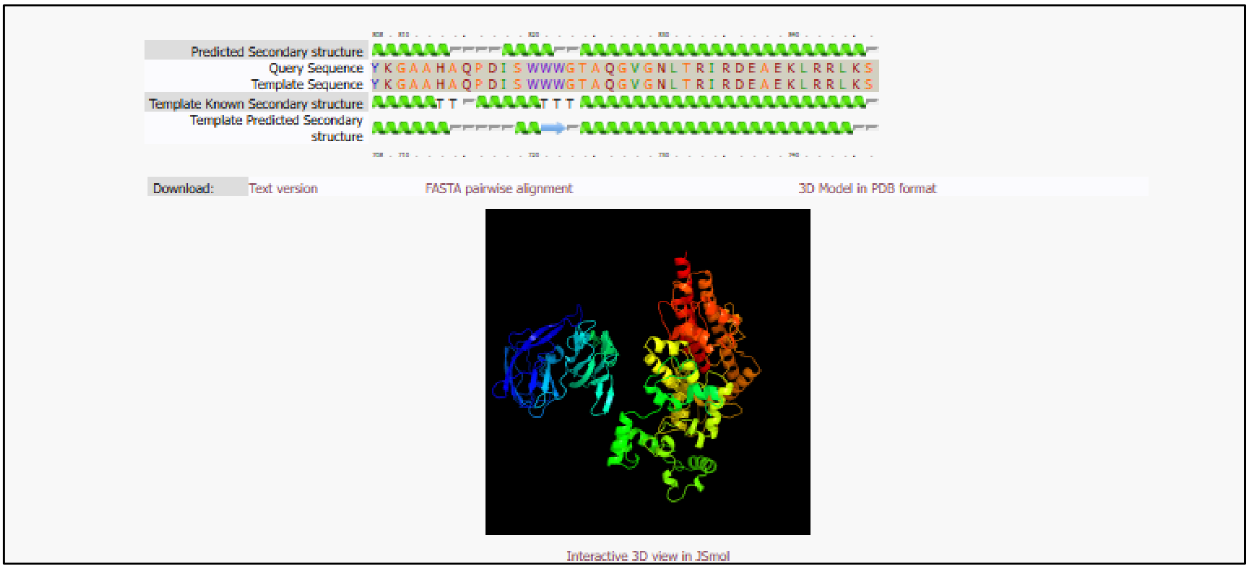

Figure S3 – Top and bottom of the output page from AlphaThread. The top part gives the AlphaFold model used as the template together with the confidence that this is an homologous template. The percent protein sequence identity for the aligned region is given. This is followed by the sequence alignment between query and template including the aligned secondary structure as in normal mode (FigureS2). At the end there is an image of the predicted secondary structure than can be viewed in Jsmol.

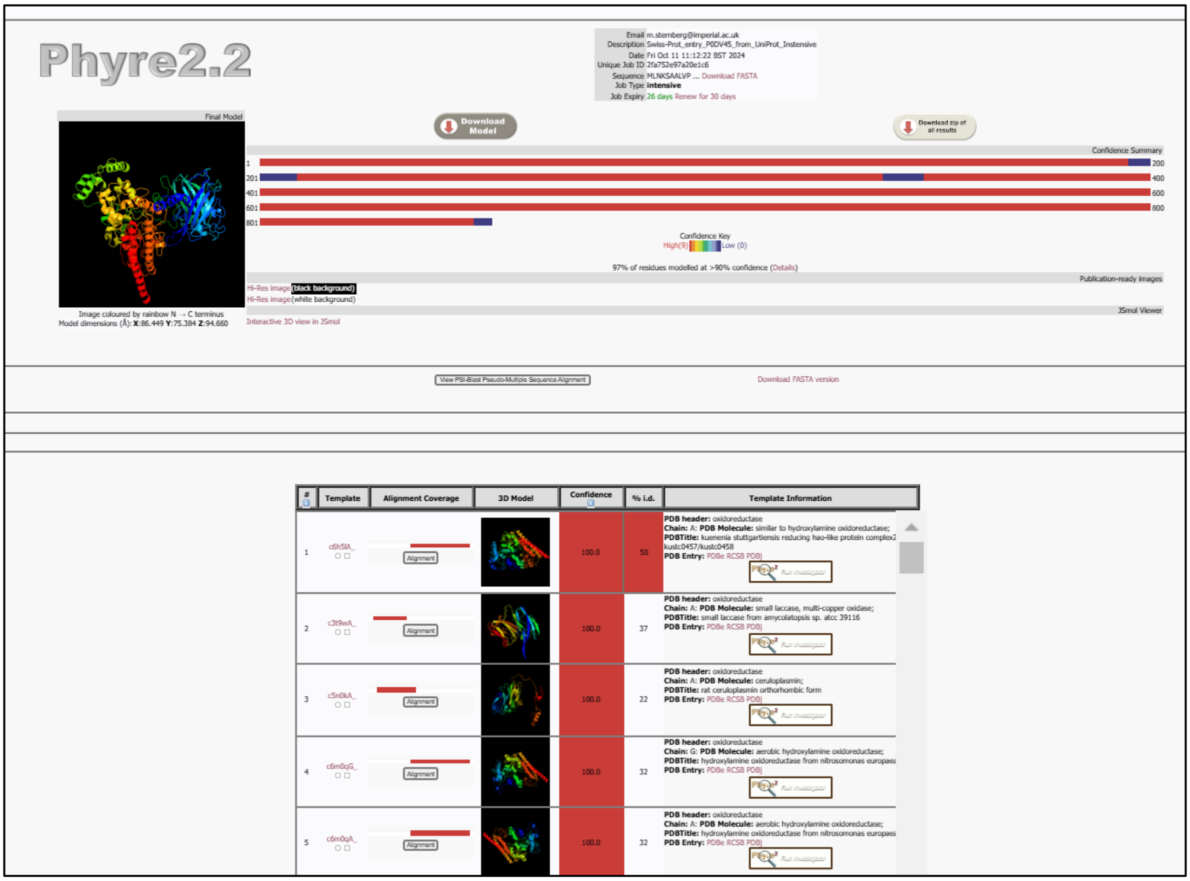

Figure S4 – Output of intensive mode. For details see main text.
